# Modular Cage-Type Prosthetic Construction for Lumbar Intervertebral Reconstruction: A Computer-Aided Design-Based Geometric Design and Finite Element Analysis Study

**DOI:** 10.7759/cureus.114416

**Published:** 2026-08-12

**Authors:** Adrian-Valentin Enache, Antonio-Daniel Corlatescu, Horia-Petre Costin, Georgian L Iacobescu, Alexandru-Vlad Ciurea

**Affiliations:** 1 Doctoral School, "Carol Davila" University of Medicine and Pharmacy, Bucharest, ROU; 2 General Medicine, "Carol Davila" University of Medicine and Pharmacy, Bucharest, ROU; 3 Neurosurgery, "Carol Davila" University of Medicine and Pharmacy, Bucharest, ROU; 4 Orthopedics and Traumatology, University Emergency Hospital, Bucharest, ROU; 5 Neurological Surgery, Sanador Clinical Hospital, Bucharest, ROU

**Keywords:** cage subsidence, computer-aided design, finite element analysis, implant optimization, interbody cage, load distribution, lumbar spine reconstruction, modular implant design, patient-specific implants, spinal biomechanics

## Abstract

Background

Lumbar interbody fusion (LIF) has become one of the most common surgical treatments for both degenerative and traumatic spinal disorders. Also, advances made in computer-aided design (CAD) and finite element (FE) modeling are creating new ways to test alternate designs and determine how they will initially behave mechanically before being tested experimentally.

Objective

We aim to create and complete a preliminary biomechanical evaluation of a new modular cage-like prosthetic construct for lumbar intervertebral reconstruction using computer-aided design (CAD) and finite element analysis (FEA).

Methods

A modular interbody prosthesis concept was created utilizing CAD with an emphasis on a stacked lamellar design and anatomically shaped contact surfaces at each endplate, and a center screw-based lock. Two different sizes of the implant were created to mimic compact and expanded constructs. FEA simulations were completed to examine stress distribution, displacement, safety factor, and load transfer characteristics of the implant under static compressive loading. Additionally, a simplified lumbar spine-cage assembly model was created to simulate implant performance in an anatomical environment.

Results

Both size configurations remained structurally intact under static compressive loading. Under standardized material, loading, contact, and meshing conditions, the 8 mm construct demonstrated a lower maximum von Mises stress than the 18 mm configuration (39.08 MPa versus 87.54 MPa), reduced global displacement (0.067 mm versus 0.122 mm), and a higher minimum safety factor (2.56 versus 1.14). The 8 mm configuration exhibited a maximum first principal stress of 55.46 MPa and a minimum third principal stress of -36.26 MPa. In the lumbar spine-cage assembly model, peak implant stress was lower than in the isolated expanded cage model, whereas global displacement increased, indicating redistribution of the applied load through the surrounding vertebral structures.

Conclusion

This proposed modular cage-like prosthetic construct demonstrated configuration-dependent mechanical properties, which enabled controlled variation in intervertebral height while maintaining the same footprint of the implant. An increase in construct height was shown to be associated with increases in localized mechanical demands. Overall, these preliminary computational results suggest the need for continued development of this modular design and additional testing via standardized biomechanical testing, experimental validation, and subsequent clinical evaluation.

## Introduction

While the restoration of disc space height and segmental lordosis are key objectives of reconstructive lumbar interbody surgery in trauma, the benefits of cages capable of expanding appear to be primarily limited to their ability to restore greater degrees of segmental lordosis and disc space height, relative to non-expanding cages. However, they do not appear to provide any benefit over static cages with regard to the restoration of global lumbar lordosis or patient-reported clinical outcomes [1-7].

Although the restoration of segmental lordosis by cages appears to be dependent upon cage lordotic angulation and the anterior location of the cage placement [1,8], and not cage material or the volume of space that the cage occupies, for both cage types, the incidence of cage collapse ("subsidence") after placement remains a major risk. This can occur due to many factors, including the degree of disc height restored, the size of the cage placed, and the condition of the bone prior to cage placement (as determined by Hounsfield units) [9,10]. There is some evidence to suggest that cages capable of expanding may experience a higher incidence of cage collapse than static cages, especially when the facets were incompletely released or a unilateral facetectomy was performed [3,4].

In addition to the effects of cage collapse, the properties of the cage material will influence the behavior of the cage construct. For example, polyetheretherketone (PEEK) cages have been shown to exhibit higher rates of subsidence and pseudoarthrosis than titanium cages [9,11-14]. Recent studies examining the effects of surface modifications of titanium cages (such as porous titanium structures and nano-etched surfaces) have shown a reduction in cage collapse and an enhancement in fusion formation, indicating that osseointegration plays a significant role in achieving long-term stability of the construct [11,12,14].

In contrast to static cages, expandable cages are typically better able to restore greater degrees of disc space height and segmental lordosis. However, the ability of expandable cages to restore overall lumbar lordosis and improve clinical-related outcomes is not universally observed, and they may have a higher incidence of cage collapse [3-7]. Conversely, while static cages tend to exhibit lower rates of cage collapse, they often provide less initial restoration of disc space height and may require additional surgical interventions to achieve adequate restoration of disc space height and/or segmental lordosis [3-5,13].

Therefore, expandable cages may be particularly useful for patients who undergo minimally invasive lateral lumbar interbody fusion (LLIF) for traumatic indications (AO Spine Type A2-A4 fractures) since this approach allows for controlled anterior column reconstruction and sagittal plane correction. A recent cohort study by Bättig et al. showed that patients who underwent treatment with expandable cages for traumatic injuries exhibited a 100% fusion rate at 12 months, a mean sagittal Cobb angle improvement of 12°, and predominantly good to excellent clinical outcomes, providing evidence for the feasibility and efficacy of using expandable cages for anterior column reconstruction and sagittal plane correction in patients with traumatic injuries [15].

Biomechanically, evidence supports the use of expandable cages as well, indicating that the larger footprint of expandable cages results in a level of stability comparable to that of anterior lumbar interbody fusion (ALIF) constructs, which could reduce the necessity of supplementary fixation in select traumatic injury scenarios [16].

Lastly, titanium cages, particularly those with modern surface features, show significantly lower rates of cage collapse and higher rates of fusion than PEEK cages in lumbar spinal fusion [9,11-14]. While the radiolucency of PEEK allows for better postoperative imaging, the relatively poor osseointegration of PEEK cages has been shown to contribute to an increased risk of cage collapse and pseudoarthrosis [9,17]. Three-dimensional (3D) printing may allow for the creation of titanium cages that provide superior outcomes compared to traditional titanium cage designs and PEEK cages through enhanced bony ingrowth and mechanical locking [11,14,18].

Lumbar lordosis restoration was identified as an independent protective factor against long-term complications [19]. Distribution of lumbosacral lordosis according to pelvic morphology is critical. Caudal and cranial contributions vary with pelvic incidence. These data support the need for patient-specific alignment strategies. Traumatic reconstruction of the lumbar spine often results in severe anatomical disruption. Precise restoration of both local segmental geometry and global spinal alignment are required for functional recovery and prevention of iatrogenic deformity [20].

Advancements in interbody cage technology have facilitated the development of expandable devices designed to achieve maximum in situ disc height restoration through minimally invasive corridors. A recent meta-analysis comparing expandable versus static cages identified nuanced tradeoffs. Expandable cages demonstrated superior disc height and foraminal height restorations than static cages. Clinical outcomes measured using patient-reported outcome measures were equivalently comparable between technologies. The higher reoperation rate observed with static cages may reflect limitations in achieving indirect decompression, while material integrity concerns under physiological loading explain the higher cage breakage observed with expandable devices [5,21-23].

Subsidence remains one of the most common postoperative complications following lumbar intervertebral fusion. The frequency of subsidence depends on surgical technique and patient factors. Frequencies range from 20% to 45%. Subsidence can result in loss of correction of disc height, recurrent foraminal stenosis, altered sagittal alignment, persistent pain, and pseudarthrosis that requires revision surgery. Systematic reviews identify poor bone quality (dual-energy X-ray absorptiometry (DEXA) T-score ≤ -1.0), age ≥ 65 years, female sex, osteoporosis, endplate injury, and overdistraction of the intervertebral space as major risk factors related to patients [9,24-26]. Narrow cage width (≤22 mm), excessive cage height (≥11 mm), posterior placement of the cage, and small footprint of the cage are identified as major risks related to the device. Dual-energy X-ray absorptiometry and its surrogate biomarkers emerge as important predictive factors. The novel MRI-based vertebral bone quality score has been shown to accurately predict subsidence in 85.6% of cases and show moderate correlation with DEXA scores (r = -0.540 to -0.546). Subsidence results from stress concentration at the interface of the cage and the endplates, which far exceeds the yield strength of trabecular bone. Subsidence ≥ 4 mm is associated with a significant increase in the incidence of pseudarthrosis [9,24,27].

The choice of materials used in cage design has a direct impact on osseointegration and subsidence risk. Although PEEK cages offer radiolucency and a Young's modulus more similar to bone than titanium, they demonstrate limited capacity for osseointegration and higher rates of subsidence than porous titanium alternatives. Recent meta-analyses compare 3D-printed porous titanium cages to PEEK cages. The comparison reveals that porous titanium cages achieved significantly higher rates of fusion at follow-up evaluations at six months and one year than PEEK cages. Only porous titanium allows for bony ingrowth through its interconnected pores [28-30]. Comparative studies using animals have demonstrated that 3D-printed porous titanium cages without autograft bone grafting outperformed PEEK cages with autologous iliac crest bone grafting in terms of osseointegration, bone-implant contact area, and osseous interdigitation at four and eight weeks after surgery. The enhanced biological performance of porous titanium stemmed from the ability to promote on-growth and in-growth simultaneously to generate biological stability through intramembranous and endochondral ossification pathways [29,30].

Geometry of the cage, including footprint, angle of lordosis, and positioning, determines biomechanical outcomes and success of clinical procedures. Multiple finite element analyses (FEA) evaluated oblique lumbar intervertebral fusions utilizing 172 different configurations. Longer cages (60 mm) were shown to reduce endplate displacements/stresses by approximately 33% versus shorter cages (40 mm). Reducing cage height from 14 to 10 mm was shown to decrease endplate stresses by approximately 53% [31]. Consistent findings indicate that anterior placement of the cage maximizes restoration of segmental lordosis. Anteriorly placed cages achieved average increases in segmental lordosis of 4.81° versus 2.47° in posteriorly placed cages at six-month follow-up evaluation. Multivariate regression analysis confirmed that cage positioning, pre-surgery lordosis, and cage height are primary determinants of post-surgical segmental lordosis (adjusted r² = 0.70) [8,32]. Angle of lordosis for the cage significantly influences alignment outcomes. Fifteen-degree lordotic cages provided better preservation/restoration of lumbar lordosis and better prevented correction loss than four-degree or eight-degree cages in transforaminal lumbar intervertebral fusions. However, each patient must optimize their own cage footprint and positioning since wider cages and anterior placement, while increasing segmental stiffness, can also increase plastic deformation and subsidence risk in patients with poor bone quality [33].

Development of modular computationally optimized lumbar interbody cage systems is motivated by recognition that individual variations in anatomy/bone structure, pathological conditions, and mechanical requirements dictate patient-specific solutions. The use of integrated computer-aided design (CAD)/finite element analysis (FEA) systems enables rapid patient-specific cage design and biomechanical evaluation that provides superior performance when compared to commercial off-the-shelf designs [34]. Application of advanced topology optimization techniques that consider the structural response of adjacent bones provides reductions in risk of subsidence of 89%-94% for titanium and PEEK materials compared to generic implants. Further, such techniques provide a 75% reduction in subsidence risk compared to mechanically non-optimized anatomically conforming implants [35]. Outputs from finite element models developed specifically for each patient provide quantitative prediction tools that include average trabecular intermediate strain (area under the curve (AUC) = 0.809) and peak endplate minimum principal stress (AUC = 0.775) that perform better than all traditional clinical metrics, including cage dimensions and bone density measurements, for predicting severe subsidence [36]. Finite element (FE) modeling provides a platform for comprehensive evaluation of materials, design parameters, fixation methods, and bone formation processes in addition to evaluating differences between fusion techniques and investigating pathological structures, capabilities that complement but cannot replace either clinical or experimental investigations alone [37]. Integration of computational modeling into the clinical workflow provides a quantitative tool for performing patient-specific preoperative planning and enables surgeons to select optimal cage geometries and placement strategies based on each patient's unique anatomic and biomechanical characteristics [33-36,38].

A prosthetic construct designed as a modular lumbar interbody cage-like device using an integrated CAD/FEA methodology provides the potential solution to address the multifactorial challenges associated with complex traumatic reconstructions of the lumbar spine. The use of a modular approach enables the optimization of cage geometry, distribution of materials, angle of lordosis, and positioning to maximize segmental lordosis restoration, minimize subsidence risk through load distribution optimization, and enhance osseointegration through surface technologies and material selection advancements. This study describes the development and validation of a modular computationally optimized lumbar interbody cage system as well as the clinical rationale supporting its development.

## Materials and methods

Computer-aided design and implant architecture

The cage-like prosthetic construct was developed using SolidWorks Premium 2020 SP3.0 (Dassault Systèmes, Vélizy-Villacoublay, France) as a computer-aided design (CAD) platform for lumbar intervertebral reconstruction. Geometric design assessment and a preliminary finite element analysis (FEA) were conducted to analyze both the structural concept of the implant and its initial mechanical behavior under simulated loading conditions. The two implant configurations were created as compact and expanded forms of 8 mm and 18 mm in height, respectively. Additionally, a simplified anatomically integrated lumbar spine-cage assembly model was evaluated in order to assess how the implant behaves when placed into a simplified vertebra-implant system. The implant geometry was developed using SolidWorks Premium 2020 SP3.0, whereas finite element simulations were performed using Autodesk Inventor 2026.

The CAD-based analysis was utilized to determine the major architectural features of the device, which include the total exterior geometry of the device, the modular arrangement of the lamellar elements, the axial locking and assembly mechanism, the geometry of the endplate-contacting surfaces, and the ability of the construct to vertically scale while maintaining the same general external footprint. From a structural standpoint, the implant is comprised of superior and inferior terminal components, intermediate modular spacers/lamellae, a central fixation pathway, and an inferior locking base. Utilizing this design method, the height of the implant can be varied in a controlled manner via modular stacking while maintaining similar terminal geometries and footprints throughout.

Finite element model configurations

Finite element modeling was used to create three simulation configurations: an isolated 8 mm cage, an isolated 18 mm cage, and an integrated lumbar spine-cage assembly. All simulations were performed using a linear static analysis approach. The isolated cage models were used to evaluate the mechanical performance of each configuration at different heights, whereas the lumbar spine-cage assembly model was intended to provide a more clinically representative assessment of load distribution between the implant and the adjacent vertebral structures.

Material properties and mesh generation

For the direct comparison of the isolated 8 mm and 18 mm cage configurations, both constructs were assigned the same homogeneous, isotropic, and linear-elastic PEEK material definition. The material properties included a mass density of 1.30096 g/cm³, a Young's modulus of 3.59906 GPa, a Poisson's ratio of 0.38, a shear modulus of 1.30401 GPa, a yield strength of 99.974 MPa, and an ultimate tensile strength of 99.974 MPa.

Identical mesh-generation parameters were applied to both isolated cage configurations. The average element size was set to 0.08 times the model diameter, the minimum element size to 0.2 times the average element size, the grading factor to 1.5, and the maximum turn angle to 60°. Curved mesh elements were not used, and part-based measures were enabled for assembly meshing. Bonded contacts were assigned between the adjacent modular components to represent a mechanically integrated construct following axial locking.

A uniformly distributed pressure of 450 psi was applied to the selected superior load-bearing surfaces of both isolated cage configurations, while the corresponding inferior supporting surfaces were assigned fixed constraints. Thus, the material definition, nominal applied pressure, contact formulation, and mesh-generation parameters were standardized between the two models.

Scope of the computational evaluation

In order to investigate how our implanted cage would behave in conjunction with surrounding bone, we created a lumbar spine-cage assembly model. Our model treated both vertebral bodies and cages as a single entity that underwent static compressive loads. The vertebral components were treated with simplified default materials having low stiffness, while the cage components were treated with PEEK material properties as previously described. We meshed our model using an average element size representing 0.1 times the diameter of our model. We set our minimum element size to 0.2 times larger than the average element size and graded at a rate of 1.5 per unit length, with no curved mesh elements permitted and allowing part-based assembly meshing. We applied a pressure load to our selected loading area of 10.000 MPa while we constrained our support surface with a combination of fixed and frictionless constraints.

Each finite element model produced in this project used several output parameters to assess structural performance during compression, including equivalent von Mises stress, first principal stress, third principal stress, total displacement, reaction force, reaction moment, and safety factor. For both our 18 mm model and our spinal-lumbar assembly model, we additionally monitored additional stress, strain, directional displacement, and contact pressures.

Our finite element analyses served as an initial biomechanical evaluation of the modular cage-like prosthetic construct developed herein. Our finite element analysis results provided initial computational data regarding stress transfer, deformation, and configuration-dependent mechanical behavior of the device upon application of compressive loads. No experimental material testing was completed, nor was any cadaveric validation, fatigue testing, or in vivo assessments made as part of this project.

## Results

**Figure 1:**
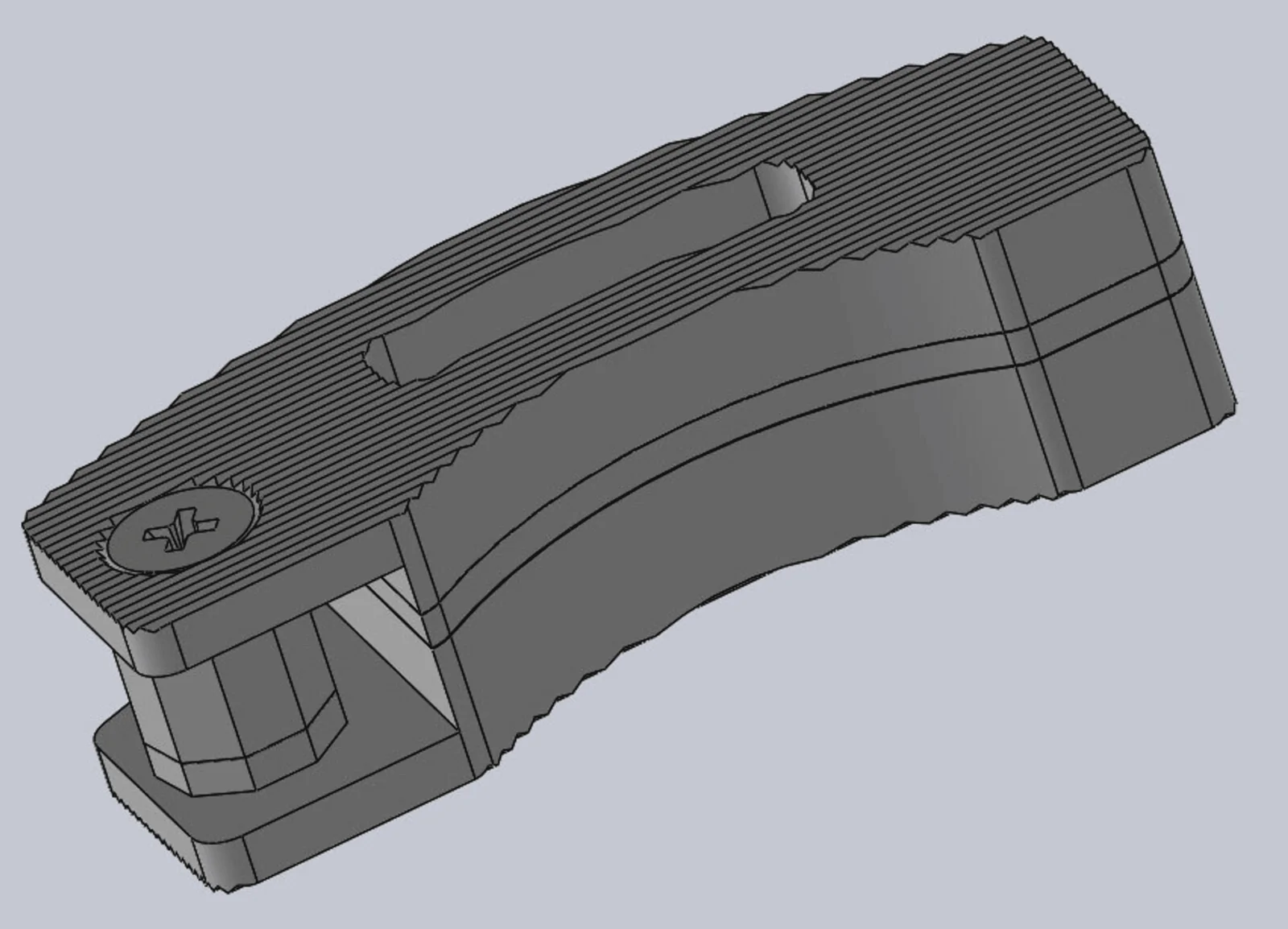
8 mm configuration of the modular cage-like prosthetic construct CAD three-dimensional representation of the entire 8 mm construct after being completely assembled. This image represents the tight configuration of the device; this includes the terminal (superior and inferior) parts of the construct, the central fixation pathway, the curved shape formed by the contact between the endplate and the lamella, and the serrations along the exterior surface of the construct, which were designed to provide additional initial mechanical stability to the implant-bone interface. CAD: computer-aided design

Modular cages are developed that provide a geometrically defined, modular, cage-like prosthetic construct intended to reconstruct lumbar intervertebral spaces. The cage has an adjustable vertical architecture with superior and inferior terminal components, a central fixation system, and various numbers of intermediate lamellar elements. With this configuration, the cage provides controlled reconstruction of the intervertebral height while providing a constant overall cage footprint. The morphology of the compact configuration (8 mm) is depicted in Figure 1.

**Figure 2:**
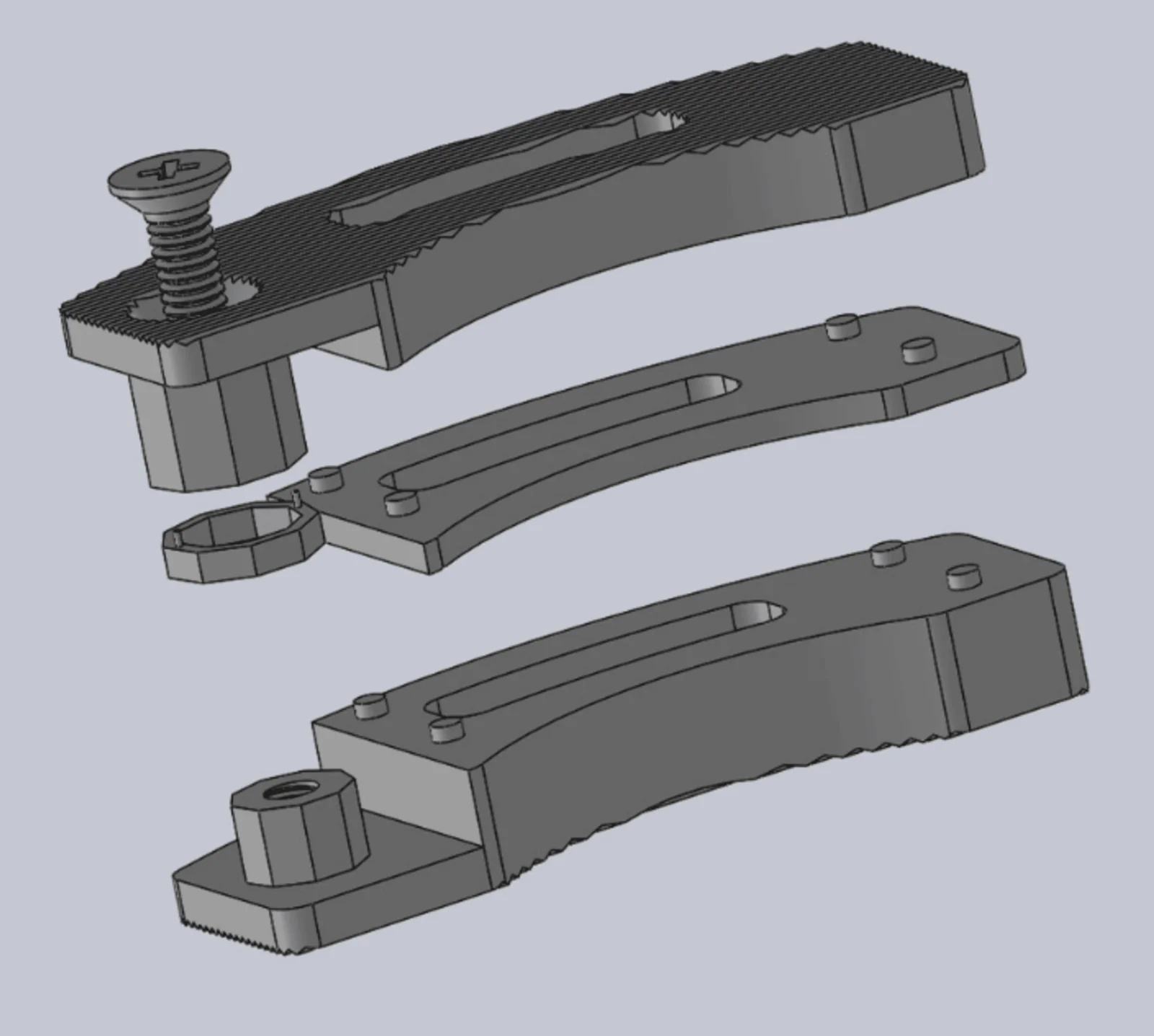
Exploded view of the 8 mm configuration of the modular cage-like prosthetic construct CAD exploded views of the 8 mm configuration represent how the individual components of the implant relate to each other. These include the superior and inferior terminal components, the lamella component located in the middle of the construct, the central fixation element consisting of a screw, and the inferior locking base. This allows to see how the individual components assemble together to achieve the desired total length of the implant and to understand the modular nature of the system. CAD: computer-aided design

The cage is constructed from two main load-bearing structures: the superior and inferior terminal components, and the intermediate modules located between the superior and inferior terminal components. The layered architecture of the cage represents the fundamental design of the cage. The cage height may be adjusted by changing the number of intermediate modules located between the superior and inferior terminal components. A modular cage design will allow the cage to accommodate a variety of disc-space sizes and maintain the same basic assembly principles and general endplate contacting geometries. The assembly process and structural organization of the thin cage are illustrated in Figure 2.

A key aspect of the cage design is the curvaceous nature of both the superior and inferior contact surfaces of the cage. These surfaces were designed to conform better to the natural curvature of the endplates of the vertebrae and to distribute loads in a manner that optimizes the cage-endplate interaction. Additionally, the exterior contact surfaces have a serrated surface texture that was designed to increase the initial mechanical grip between the cage and the surrounding bone and to resist cage migration. These characteristics will assist in establishing an immediate position of stability of the cage and provide a greater degree of mechanical engagement in the intervertebral space.

**Figure 3:**
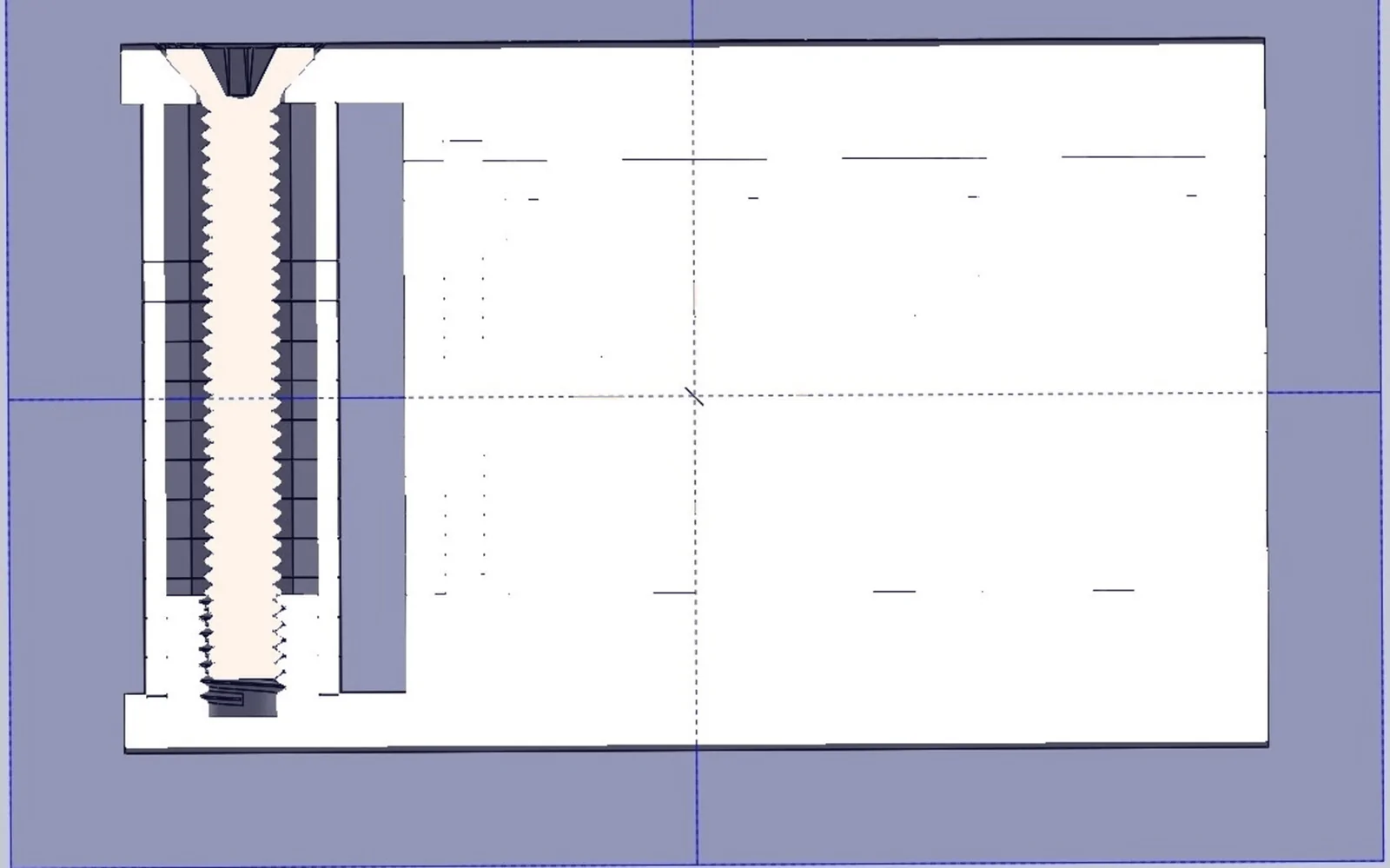
Sectional view of the 18 mm configuration of the modular cage-like prosthetic construct CAD sectional views of the 18 mm configuration represent the internal fixation architecture of the constructed 18 mm configuration. The image provides a detailed illustration of the countersunk screw entry in the superior portion of the construct, the central axial fixation pathway, the internal stacked lamellar elements that make up the module, and the threaded inferior locking base, which compresses the assembled construct into a single rigid piece of hardware. CAD: computer-aided design

The interior of the cage consists of a central screw-driven locking mechanism. The superior terminal component includes an elongated hole and a countersunk hole at the entry point for the fixation element, which travels through the center of the cage and engages a threaded receiving hole located in the inferior terminal component. Upon tightening of the fixation element, it will compress the layers of the cage together, creating a mechanically integrated cage from the previously separate elements. Therefore, the locking mechanism will create a rigid assembly of all of the components of the cage. The mechanism of the locking system and the path of axial compression are illustrated in Figure 3.

**Figure 4:**
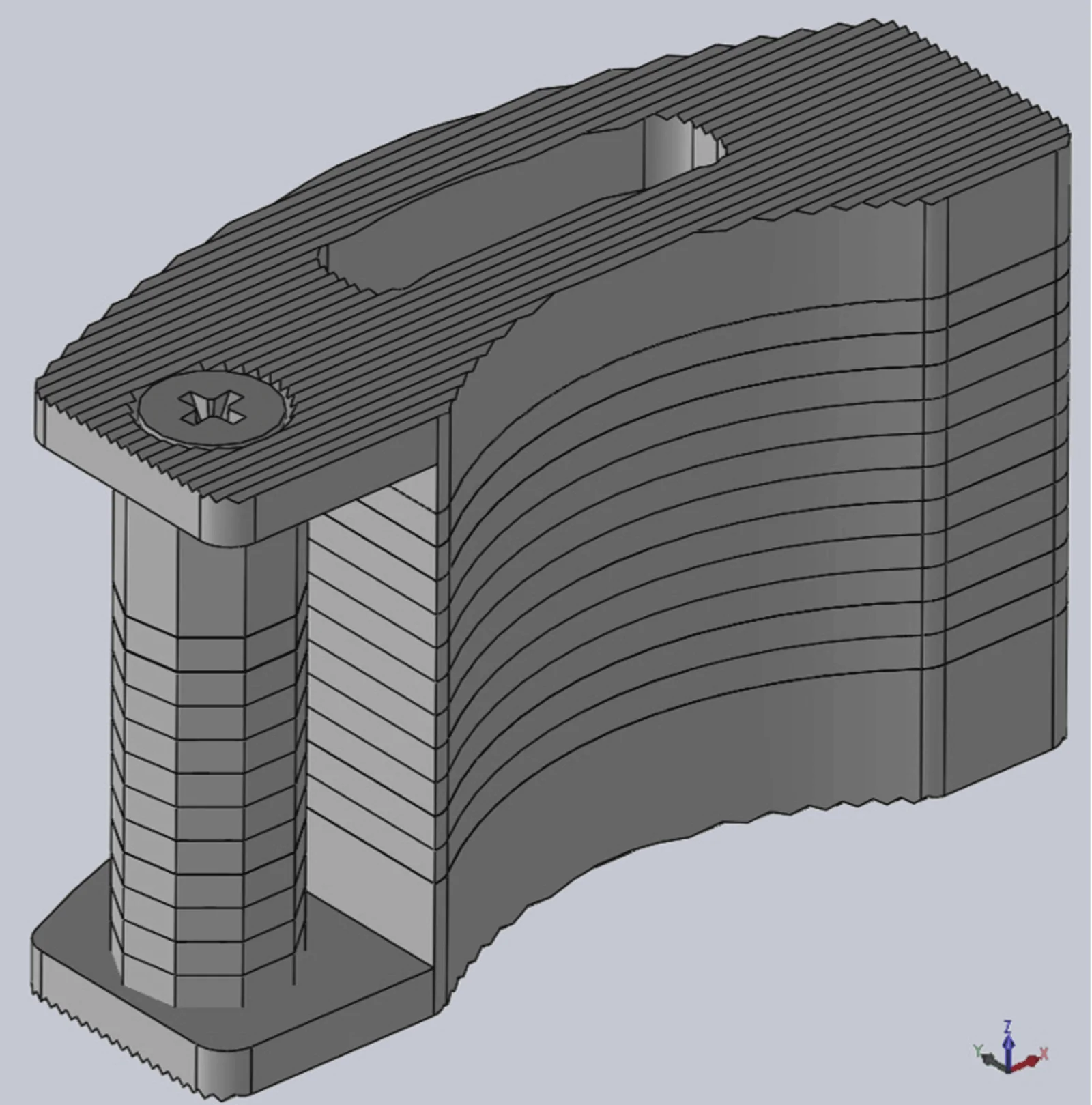
18 mm configuration of the modular cage-like prosthetic construct Three-dimensional CAD representations of the complete assembled 18 mm configuration. Compared to the 8 mm configuration, the increased height is due to an increased number of internal lamellar modules stacked on top of one another while maintaining the same terminal configuration and geometry, the curved exterior contour, and serrated endplate-contacting surface. CAD: computer-aided design

Designations of different module sizes (e.g., 8 mm versus 18 mm) provide evidence for the versatility of this modular design approach. A smaller version of this design (the 8 mm) has fewer intermediate layers; therefore, it can accommodate smaller disc spaces or where less reconstruction is required. On the other hand, a larger version of the design (the 18 mm) has many additional intermediate lamellar modules and thus provides greater vertical expansion; however, it retains the same top and bottom plate geometry. Therefore, it is evident that, through internal modular extension, one can control the height of the implant, not necessarily its footprint. In Figure 4, the fully assembled larger size construct is illustrated.

**Figure 5:**
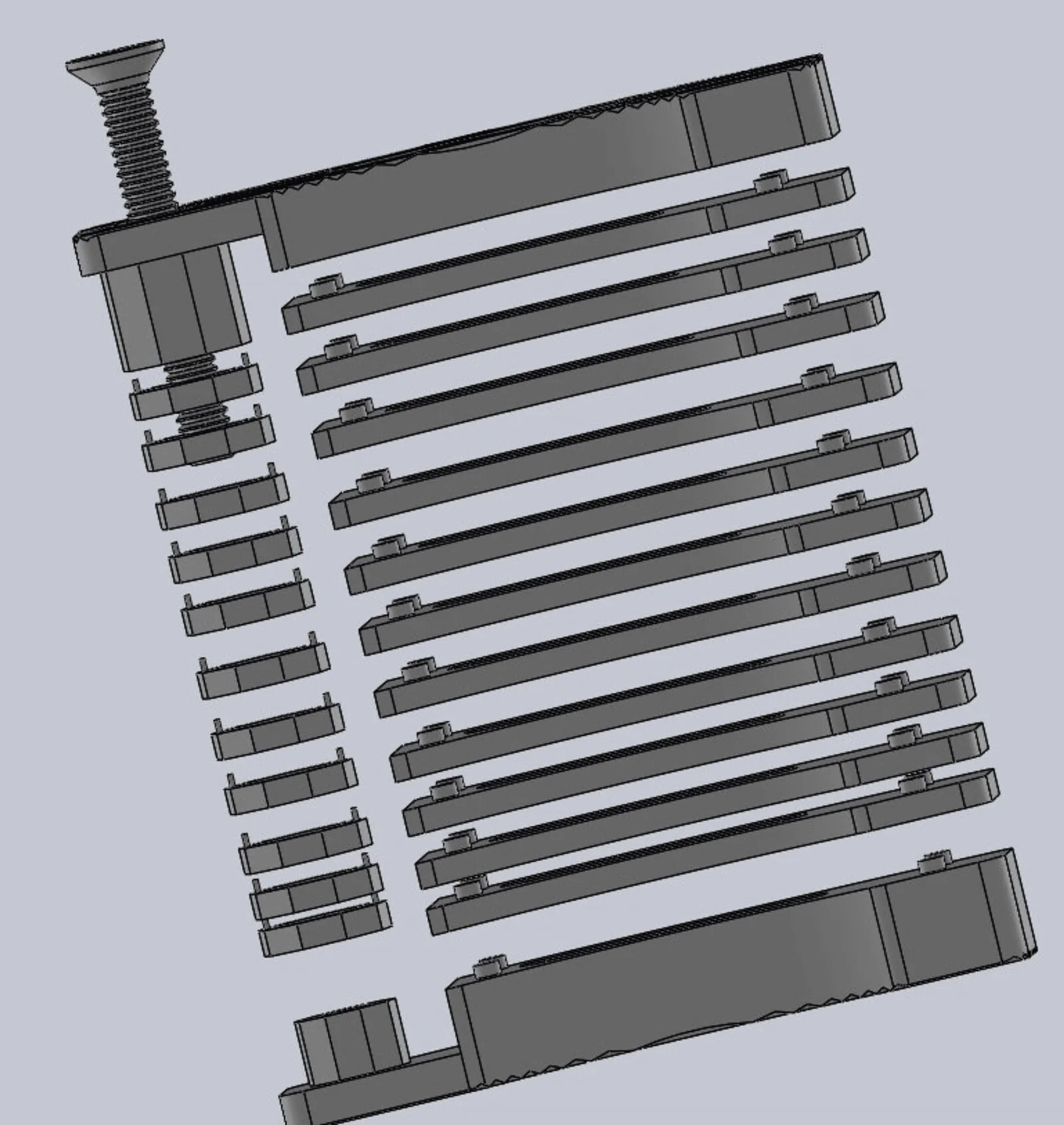
Exploded views of the 18 mm configuration of the modular cage-like prosthetic construct CAD exploded representations of the 18 mm configuration demonstrate the multi-layered modular architecture of the device. The images illustrate the terminal (superior and inferior) parts of the construct, the repeated intermediate lamella spacers, the features that allow the spacers to be aligned vertically to form a stable stack, the inferior locking base, and the central screw that is responsible for compressively loading the stacked construct to form the final assembly. When taken together, these views illustrate the method of increasing the height of the implant while minimizing the overall implant footprint through a stackable design approach. CAD: computer-aided design

The intermediate lamellar modules have the same longitudinal curvature as the terminal plates and are therefore continuous geometrically throughout the entire assembly. The intermediate modules serve two functions; they assist in providing a means of adjusting the height of the implant, and they participate in the load-bearing capabilities of the entire device. In addition, several of the intermediate modules include alignment aids such as posts and corresponding recesses that allow for accurate position registration between adjacent modules. Through the use of these interlocking features, the precision of the assembly process is improved, and the potential for undesired translation movement of the individual modules is limited, resulting in increased stability of the final assembly. The stacking architecture of the 18 mm construct is shown in detail in Figure 5.

Structurally, the device is designed as a rigid cage-like prosthesis once locked. After assembly, the forces generated are transmitted from the terminal surfaces and through the internally stacked architecture. The continuity of the side walls and a consistent outer contour further enhance the concept of the mechanically unitized interbody implant. The primary focus of this design is to restore segmental height and stabilize the reconstructed disc space through structural support.

The configuration of the sectioned construct further illustrates how the path of the fixation screw was integrated with the modular body of the implant. The central screw axis, located at the superior aspect of the device and extending downward into the locking base, serves as an assembly tool and also serves as a compressive axis and structural axis to provide cohesive mechanical strength. As a result, this construct integrates modular expandability with a definite locking mechanism, providing an architecture that is both custom and mechanically solid upon completion of the final tightening sequence.

In summary, this design approach embodies three key design concepts: restoration of intervertebral height through stackable internal modules, secure assembly of the modules via axial screw-based locking, and enhancement of the contact area at the interfaces between the implant and the vertebral bodies through the use of serrated surfaces and anatomically contoured terminal components. These design concepts collectively define the construct as a modular cage-like prosthetic system for lumbar interbody support, designed to provide the combination of geometrical flexibility and structural integrity.

Since the present study utilized only CAD renderings, the above description should be considered as a geometric and conceptual representation of the device, rather than as a biomechanical evaluation.

Finite element model of the 8 mm cage construct

A static finite element analysis was performed on the assembled 8 mm cage configuration using the standardized PEEK material definition and a uniformly distributed pressure of 450 psi. The model had a volume of 1451.15 mm³ and a calculated mass of approximately 0.00189 kg. The applied loading generated a total reaction force of approximately 472.0 N at the fixed constraint, with the principal reaction-force component occurring along the primary loading axis.

**Figure 6:**
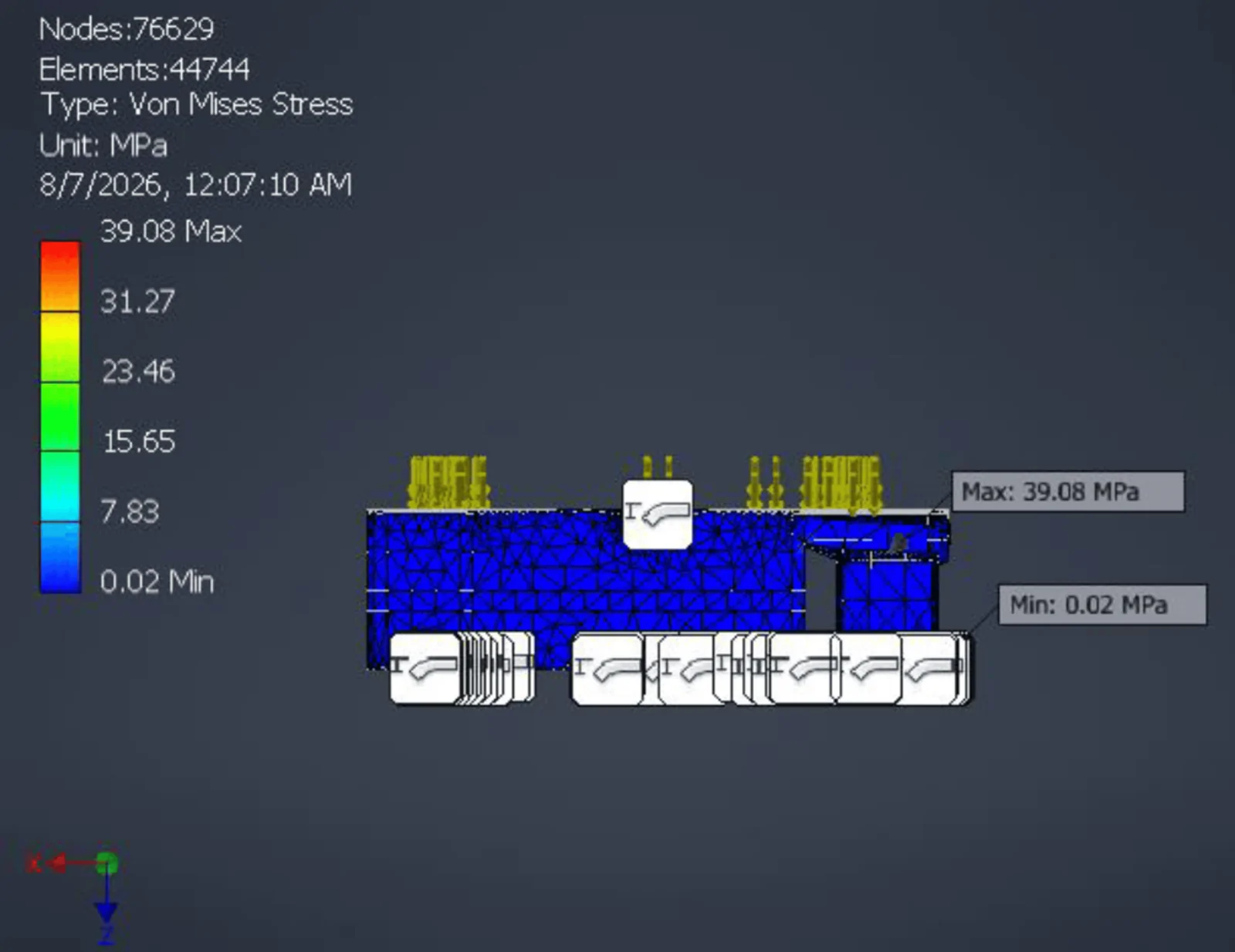
Von Mises stress distribution in the isolated 8 mm modular cage configuration under standardized compressive loading Finite element contour plot illustrating the equivalent von Mises stress distribution in the assembled 8 mm modular cage subjected to a uniformly distributed pressure of 450 psi. The model was assigned standardized PEEK material properties and bonded contacts between adjacent modular components. Most of the construct remained within a low-to-moderate stress range, while localized stress concentrations were observed in the principal load transfer and geometric transition regions. The maximum recorded von Mises stress was 39.08 MPa, remaining below the assigned PEEK yield strength of 99.97 MPa. PEEK: polyetheretherketone

The equivalent von Mises stress ranged from 0.021 MPa to 39.082 MPa. The first principal stress ranged from -10.848 MPa to 55.462 MPa, whereas the third principal stress ranged from -36.257 MPa to 27.118 MPa. The stress distribution demonstrated predominantly low-to-moderate values throughout the construct, with localized stress concentrations in the principal load transfer and geometric transition regions (Figure 6).

**Figure 7:**
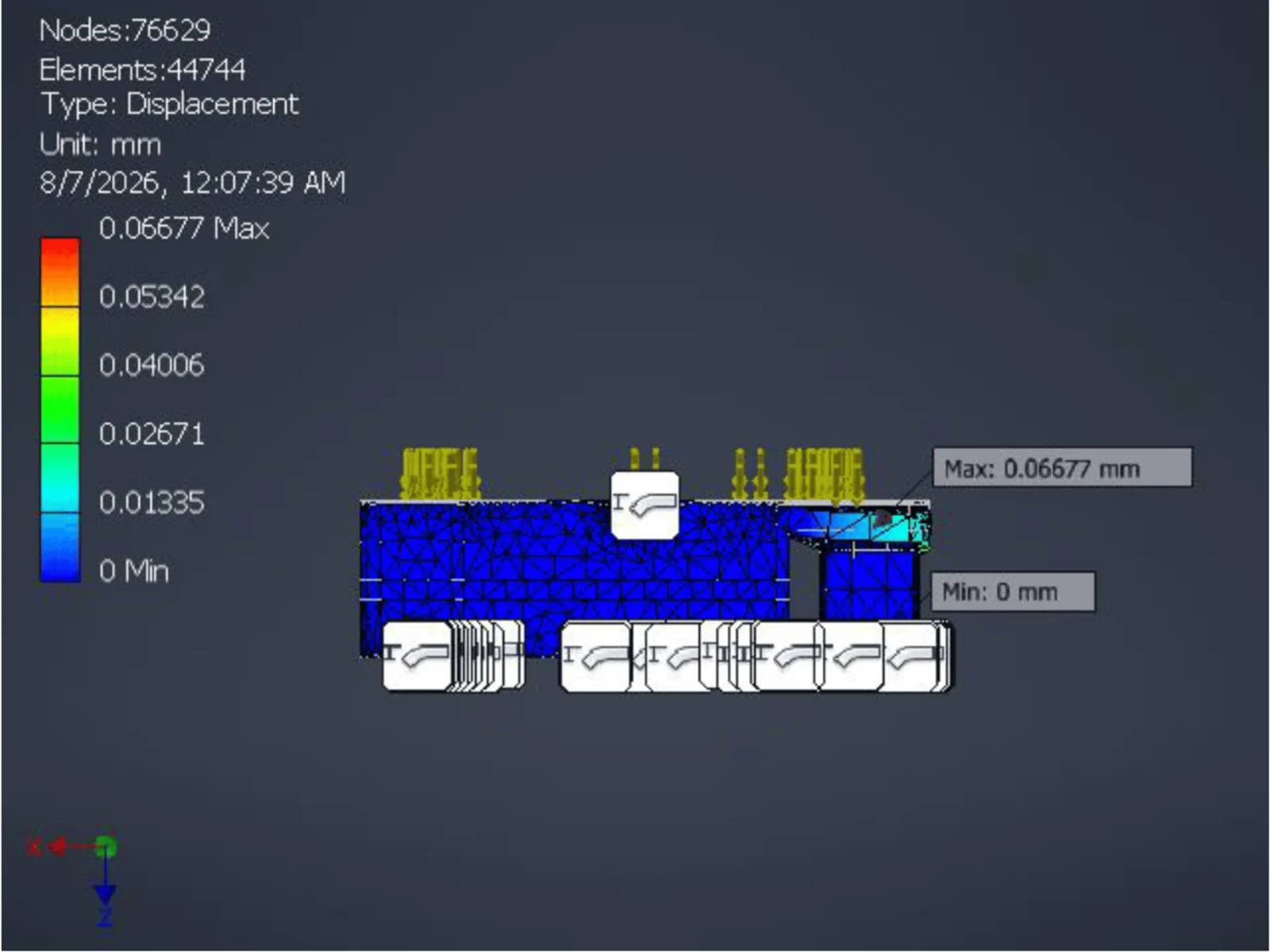
Total displacement distribution in the isolated 8 mm modular cage configuration under standardized compressive loading Finite element contour plot illustrating the total displacement distribution in the assembled 8 mm modular cage subjected to a uniformly distributed pressure of 450 psi. The displacement pattern remained limited throughout most of the construct, with the maximum deformation localized near the loaded and geometrically transitional regions. The maximum total displacement was 0.06677 mm, indicating a relatively stiff global response of the compact configuration under the simulated loading conditions.

The maximum total displacement was 0.06677 mm, while the minimum displacement was 0 mm at the constrained surfaces. The predominant directional displacement occurred along the principal loading axis. The calculated safety factor ranged from 2.558 to 15, indicating that the maximum equivalent stress remained below the yield-strength threshold defined in the material model (Figure 7).

Contact analysis demonstrated a maximum contact pressure of 139.218 MPa. The localized contact pressure concentrations were principally associated with the interfaces and geometric regions involved in transferring load between the modular components. Overall, the 8 mm configuration preserved structural continuity under the simulated static loading conditions and demonstrated limited global deformation. Although localized stress and contact pressure concentrations were present, the minimum calculated safety factor remained above unity.

Finite element analysis of the 18 mm cage configuration

A preliminary finite element analysis was run on the fully constructed 18 mm cage assembly during static compressive loading. The constructed physical model weighed 0.00386053 kg. It also had a total surface area of 6643.15 mm² and a total volume of 2946.97 mm³. In the simulation environment, it was found that the simulated volume was 2790.07 mm³, and the simulated weight was 0.00362975 kg. The assembly was assigned PEEK material properties. Simulations were run using meshes that corresponded to approximately 8% of the diameter of the modeled part. A pressure load of 450 psi was placed onto the selected load-bearing surface, while a fixed boundary condition was established at the opposite boundary condition. Bonded contacts were created throughout all interfaces within the modular assembly.

In addition to generating a reaction force magnitude of 645.543 N at the constrained region, there was also a significant reaction force component along the z-axis (645.531 N). Therefore, the primary response mechanism of the assembly was through axial compression. Additionally, the reaction moment magnitude was 0.498342 N·m.

**Figure 8:**
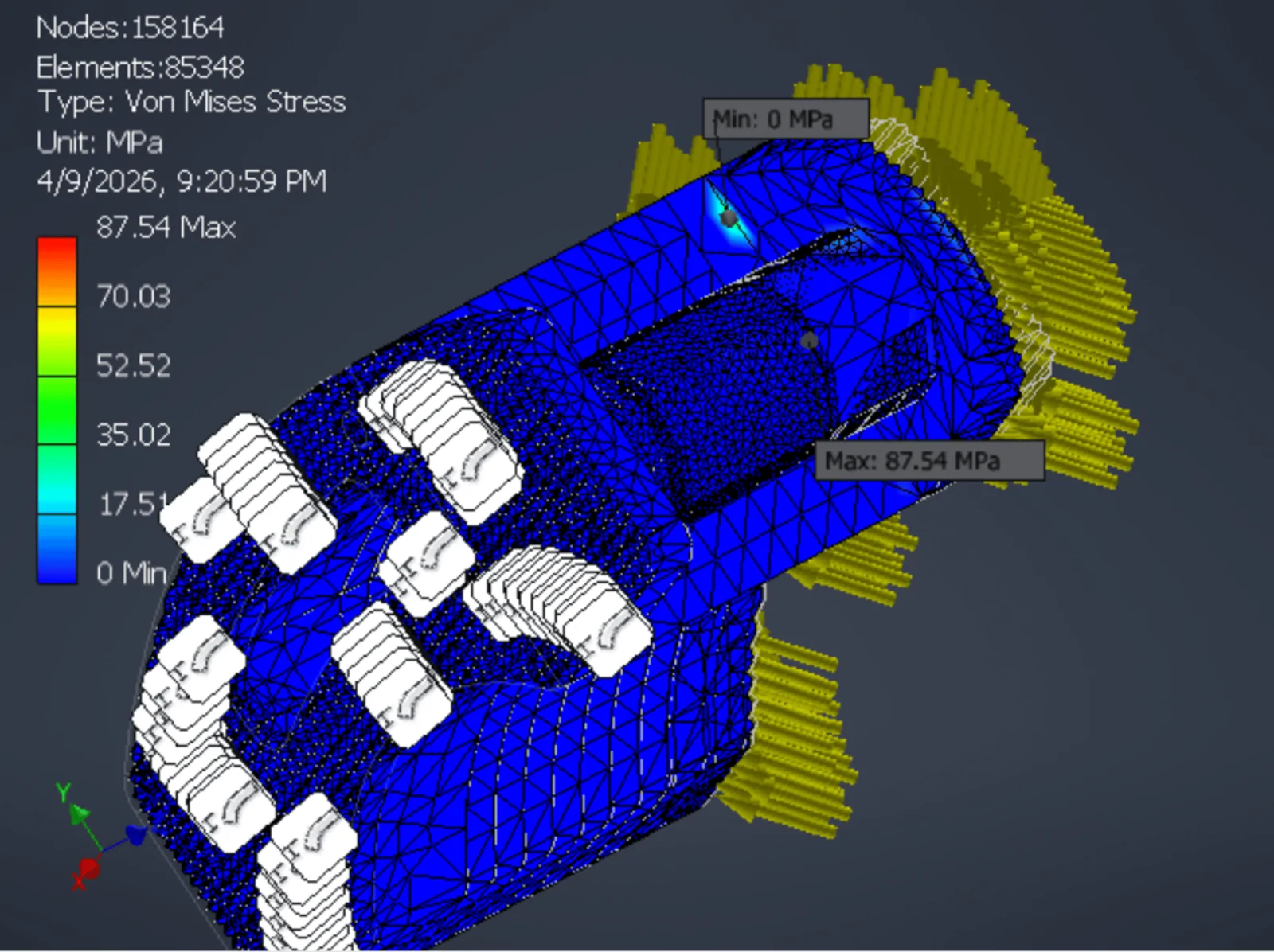
Von Mises stress distribution in the isolated 18 mm cage configuration under compressive loading Finite element contour plot illustrating the equivalent von Mises stress distribution in the assembled 18 mm modular cage configuration subjected to compressive loading. The stress map demonstrates that most regions of the construct remained within a relatively low stress range, while pronounced stress concentration was observed near the superior opening and adjacent geometric transition zone, corresponding to the principal load transfer region. The maximum recorded von Mises stress was 87.54 MPa, indicating a more mechanically demanding response of the expanded 18 mm configuration compared with the compact construct under the simulated loading conditions.

The maximum equivalent von Mises stress was 87.5377 MPa, but this value occurred over a small range in comparison to no stress occurring elsewhere in the model. The first principal stress values ranged from -13.4489 MPa to 55.7971 MPa; therefore, some of the models experienced tensile loading, while other areas experienced compressive loading. Likewise, the third principal stress values ranged from -67.425 MPa to 13.993 MPa; therefore, the 18 mm configuration experienced a biaxial/mixed stress state with both tensile and compressive loads present in different parts of the assembly. Stress contour plots showed that most of the peak stresses are located primarily at the superior opening, as well as in proximity to geometric transitions throughout the entire length of the upper portion of the assembly (Figure 8).

The maximum total displacement recorded in the model was 0.122139 mm, while the minimum displacement was 0 mm at the constrained region. Directional displacement analysis showed a maximum X displacement of 0.00639227 mm, a maximum Y displacement of 0.027041 mm, and a maximum Z displacement magnitude of 0.11661 mm, indicating that the principal deformation occurred along the loading axis. Equivalent strain reached a maximum value of 0.022385, while the first principal strain ranged up to 0.0193366, and the third principal strain reached a minimum of -0.0193882.

The calculated safety factor ranged from 1.14207 to 15. Although most of the construct remained within a favorable range, the minimum safety factor was localized to the same superior geometric transition region in which peak von Mises and principal stresses were observed. This pattern suggests that the mechanically most demanding area of the 18 mm construct is located near the load transfer region at the superior aspect of the implant.

**Figure 9:**
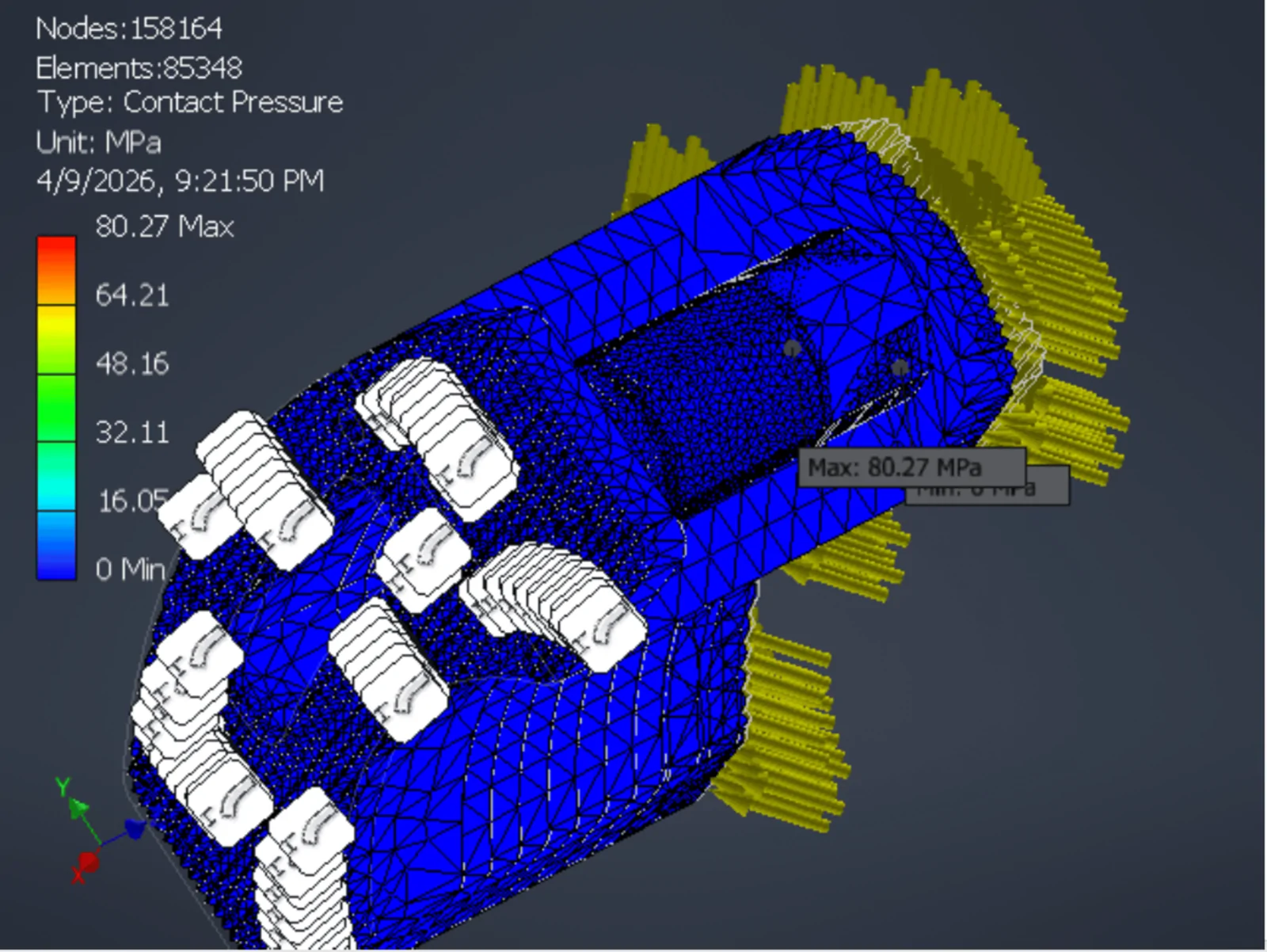
Contact pressure distribution in the isolated 18 mm cage configuration under compressive loading Finite element contour plot illustrating the overall contact pressure distribution in the assembled 18 mm modular cage configuration subjected to compressive loading. The map shows that contact stresses were concentrated predominantly in the superior load transfer region and adjacent interface zones, whereas most of the remaining constructs exhibited low contact pressure values. The maximum recorded contact pressure was 80.27 MPa, indicating substantial localized interface loading within the expanded 18 mm configuration under the simulated loading conditions.

Contact analysis demonstrated a maximum contact pressure of 80.2681 MPa. The contact pressure components ranged from -22.0039 MPa to 34.1508 MPa in the X direction, from -13.0601 MPa to 15.7975 MPa in the Y direction, and from -66.9579 MPa to 73.1162 MPa in the Z direction. These values indicate that load transfer through the construct was associated with substantial localized contact stresses, particularly along the primary compression axis (Figure 9).

Overall, the finite element findings suggest that the 18 mm modular cage configuration preserved structural continuity under the simulated compressive load and distributed the applied forces through the integrated modular body of the implant. However, compared with a more compact configuration, the increased vertical span of the 18 mm model was associated with higher peak stress values, greater overall displacement, and a lower minimum safety factor, indicating a mechanically more demanding loading response in the expanded configuration.

Comparative analysis

Under standardized material properties, nominal applied pressure, contact definitions, and mesh-generation parameters, both the 8 mm and 18 mm cage configurations maintained structural continuity under static compressive loading. The compact 8 mm configuration demonstrated a lower maximum von Mises stress and lower total displacement than the expanded 18 mm configuration. The maximum von Mises stress was 39.0819 MPa in the 8 mm construct and 87.5377 MPa in the 18 mm construct, while the maximum total displacement was 0.0667708 mm and 0.122139 mm, respectively.

The minimum safety factor was 2.55806 for the 8 mm configuration and 1.14207 for the 18 mm configuration. Under the simulated nominal pressure conditions, the compact construct therefore remained farther below the assigned material yield threshold than the expanded construct.

The maximum first principal stress was similar between the two configurations, reaching 55.4623 MPa in the 8 mm model and 55.7971 MPa in the 18 mm model. However, the expanded configuration demonstrated a greater compressive third principal stress, with a minimum value of -67.425 MPa compared with -36.257 MPa in the compact configuration. These findings indicate a more pronounced localized compressive stress response in the expanded construct.

Although an identical nominal pressure of 450 psi was applied to both models, the pressure acted over configuration-specific selected surfaces. Consequently, the total reaction forces differed between the simulations, reaching approximately 472.0 N in the 8 mm model and 645.543 N in the 18 mm model. The results should therefore be interpreted as a comparison under standardized nominal pressure rather than as a force-normalized assessment of geometry alone.

Overall, the 18 mm configuration maintained its basic load-carrying continuity but demonstrated higher equivalent stress, greater displacement, a lower minimum safety factor, and a more pronounced compressive stress response than the compact 8 mm configuration. These findings suggest that the increased vertical span and number of modular components were associated with a more mechanically demanding response under the simulated nominal pressure conditions (Table 1).

**Table 1:** Comparative biomechanical profile of the 8 mm and 18 mm cage configurations

Outcome	8 mm cage	18 mm cage
Maximum von Mises stress (MPa)	39.0819	87.5377
Maximum 1st principal stress (MPa)	55.4623	55.7971
Minimum 3rd principal stress (MPa)	-36.257	-67.425
Maximum displacement (mm)	0.0667708	0.122139
Minimum safety factor	2.55806	1.14207
Reaction force (N)	472.0	645.543
Maximum contact pressure (MPa)	139.218	80.2681

Finite element analysis of the lumbar spine-cage assembly

A preliminary finite element analysis was conducted on a lumbar spine-cage assembly under static compressive loading. This is to provide insight into how the mechanical response of the lumbar spine would respond when the implant was placed in an anatomical configuration as opposed to a stand-alone implant.

The models consisted of the cage construct located between two vertebrae. The pressure applied to the top surface of the cage was 10.00 MPa. The total weight of the model assembly was 0.0309044 kg. The total surface area of the model assembly was 35.5318 mm². The volume of the model assembly was 138893 mm³.

In addition to the above data, within the FEA software package, the calculated mass of the model assembly was 0.0309579 kg. The calculated volume of the model assembly was 138890 mm³.

Meshing parameters were defined so that an average element size equated to approximately 0.1 times the diameter of the model. Additionally, it was required that all elements be no smaller than 0.2 times the average element size. Also, there was a requirement for an element grading ratio of 1.5. However, this requirement may have been violated since an element grading ratio of 1.5 does not guarantee that the smallest element will meet or exceed 0.2 times the average size. Finally, the maximum turn angle allowed by the mesh-generation algorithm was set at 60°.

Material properties for the vertebral components were set to a low-stiffness default material. Material properties for the cage constructs were set to PEEK. Boundary conditions for the analysis were specified as follows. The bottom-most surfaces of each vertebral component were constrained such that they did not move laterally and could only displace vertically downward due to vertical displacement caused by the applied loads. The uppermost surfaces of each vertebral component were constrained such that they could freely displace in three-dimensional space but could not rotate about any axes. It was assumed that these boundary conditions simulated support provided to the model assembly during testing. The applied vertical load produced a resultant reaction force magnitude of 7239.98 N at both constraint types. In general terms, the majority of the reaction forces developed at the constraints acted in the vertical direction. Specifically, the Y-directional component of the reaction force at each type of constraint averaged around 7218.56 N, whereas the Z-directional component of the reaction force at each constraint averaged around -556.49 N. Thus, the most significant component of the reaction forces resulted from vertical movement of the model assembly. The X-directional component of the reaction force at each type of constraint was negligible. As mentioned previously, a reaction moment developed in addition to reaction forces and its magnitude was found to be 25.14 N-m.

**Figure 10:**
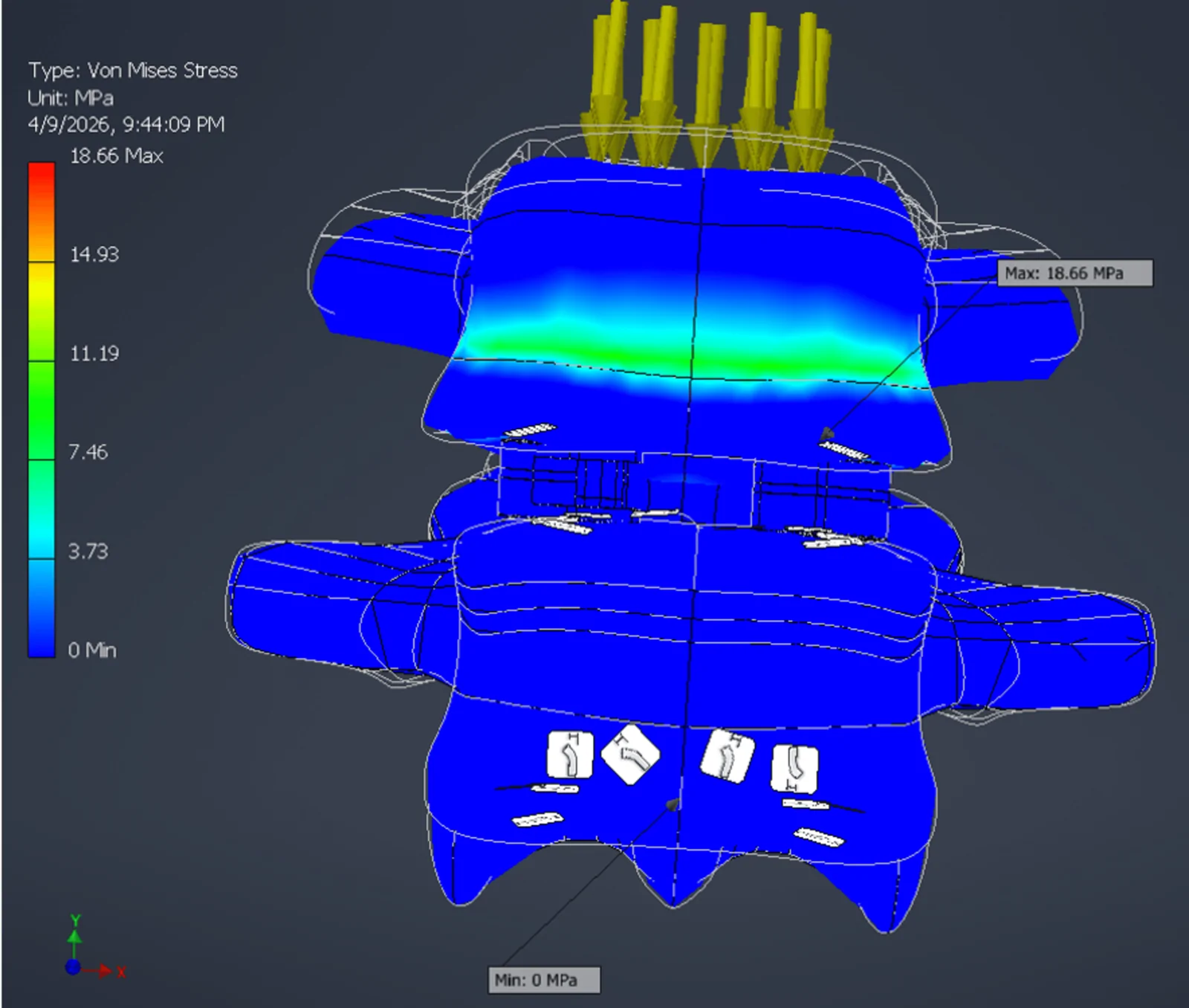
Von Mises stress distribution in the lumbar spine-cage assembly under compressive loading Finite element contour plot illustrating the equivalent von Mises stress distribution in the lumbar spine-cage assembly subjected to compressive loading. The stress map shows that the highest stresses were concentrated predominantly in the superior vertebral region adjacent to the implant interface, while the remainder of the construct remained within a relatively low stress range. The maximum recorded von Mises stress was 18.66 MPa, indicating that, within the integrated vertebra-implant model, the applied load was redistributed across the surrounding spinal structures rather than being concentrated exclusively within the cage itself.

Therefore, it can be concluded based upon results obtained from this analysis that within the context of the spinal column-implant assembly, the cage construct underwent deformation primarily resulting from combined axial and bending deformations as opposed to purely axial compression. Maximum von Mises stresses calculated throughout this analysis varied from zero to a value of 18.66 MPa. First principal stresses varied from -4.84 MPa to 9.80 MPa throughout this analysis. Third principal stresses varied from -24.58 MPa to 0.55 MPa throughout this analysis (Figure 10).

**Figure 11:**
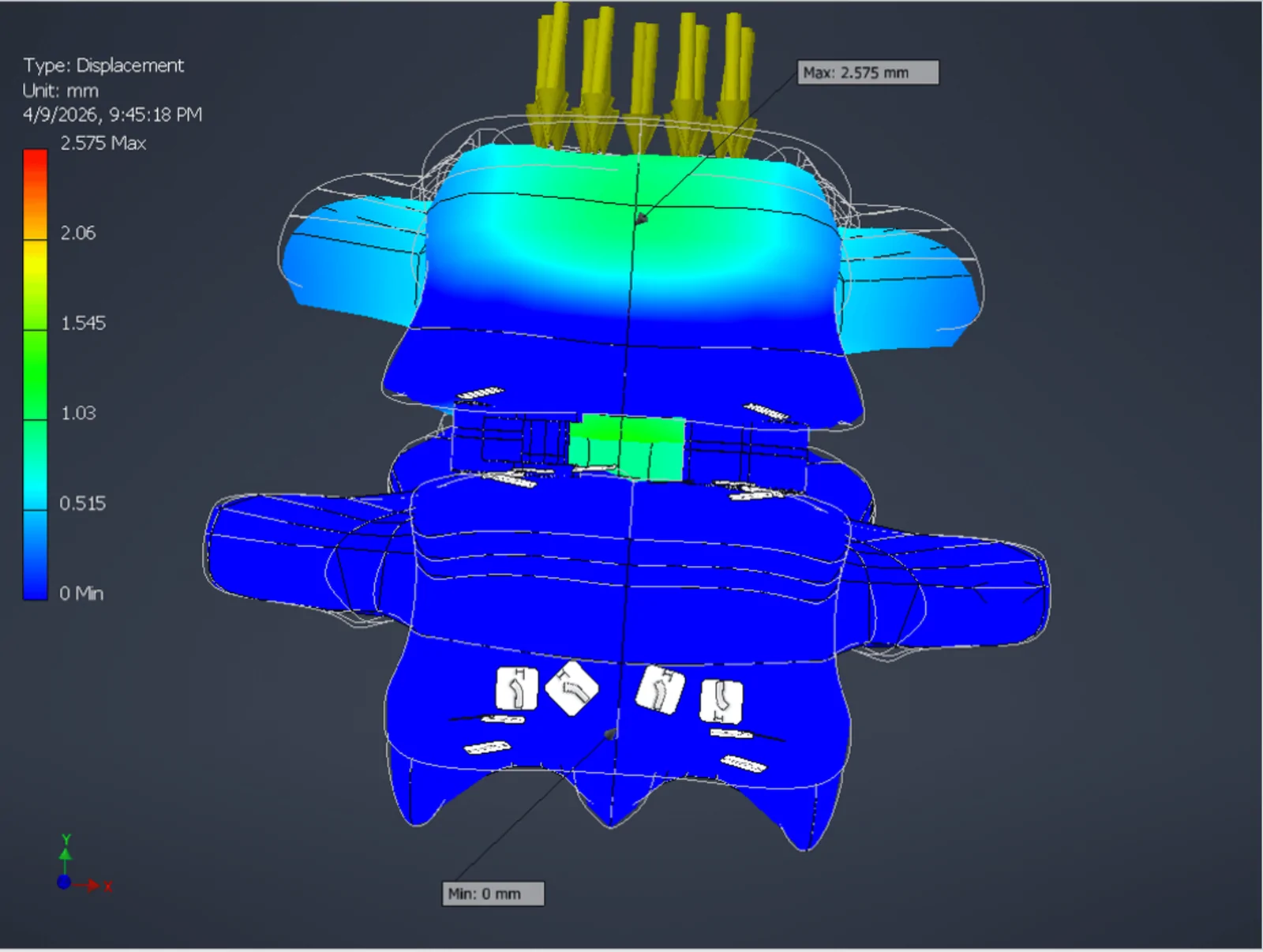
Total displacement distribution in the lumbar spine-cage assembly under compressive loading Finite element contour plot illustrating the total displacement distribution in the lumbar spine-cage assembly subjected to compressive loading. The displacement map shows that deformation was concentrated predominantly in the superior vertebral component, whereas the inferior vertebral region and adjacent portions of the construct remained within a relatively low displacement range. The maximum recorded total displacement in this plot was 2.57539 mm, indicating substantial global deformation of the integrated vertebra-implant model under the simulated loading conditions.

The maximum total displacement recorded in the model was 2.57539 mm, with a minimum value of 0 mm. Directional displacement analysis demonstrated a maximum X displacement of 0.513888 mm, a minimum Y displacement of -2.56422 mm, and a minimum Z displacement of -0.507668 mm. This displacement pattern suggests that deformation occurred primarily along the principal loading direction, with smaller associated transverse components reflecting global construct deformation under compressive loading (Figure 11).

Equivalent strain reached a maximum value of 0.181677. The first principal strain ranged up to 0.101705, whereas the third principal strain reached a minimum of -0.221518. These strain values indicate that the vertebra-cage assembly underwent substantially greater overall deformation than the isolated cage models, which is consistent with the inclusion of the surrounding spinal structures and their lower stiffness relative to the implant itself.

**Figure 12:**
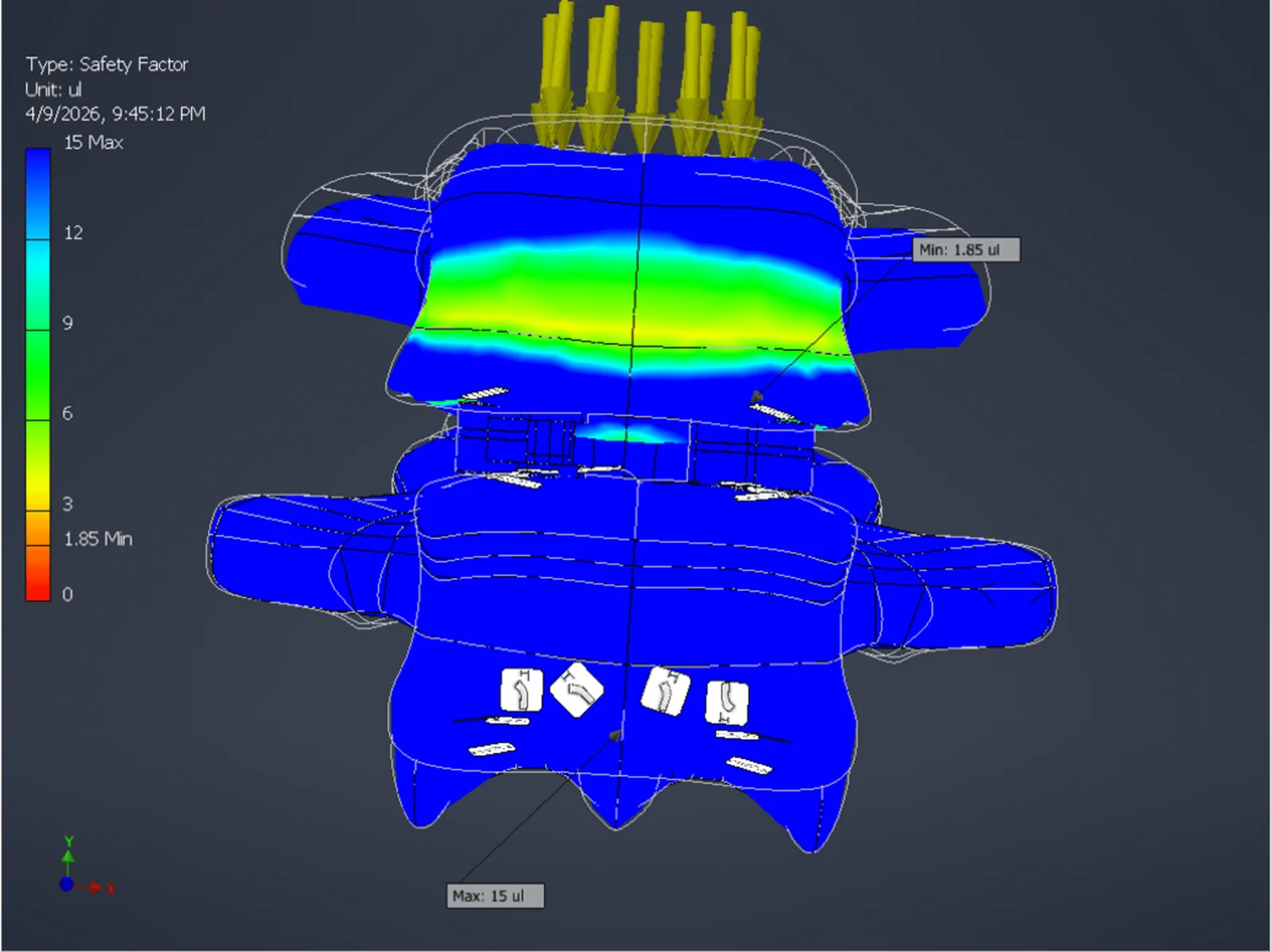
Safety factor distribution in the lumbar spine-cage assembly under compressive loading Finite element contour plot illustrating the safety factor distribution in the lumbar spine-cage assembly subjected to compressive loading. The map shows that most regions of the construct remained within a favorable safety range, while lower safety factor values were concentrated predominantly in the superior vertebral region adjacent to the implant interface, corresponding to the principal load transfer zone. The minimum recorded safety factor was approximately 1.85, indicating that the integrated vertebra-implant construct remained within the defined material strength limits under the simulated loading conditions, although with localized mechanically more demanding regions.

The calculated safety factor ranged from 1.84776 to 15, indicating that, under the simulated loading conditions, the model remained within the allowable range defined by the material properties, although the lower safety factor values suggest the presence of localized mechanically demanding regions within the construct (Figure 12).

Overall, the finite element findings suggest that the lumbar spine-cage assembly preserved structural continuity under the applied compressive load and allowed load transfer through both the implant and the adjacent vertebral structures. Compared with the isolated cage models, the integrated assembly demonstrated lower peak von Mises stress within the implant region but substantially greater global displacement, indicating that the surrounding vertebral components redistributed the load while also contributing to increased overall construct compliance.

## Discussion

Restoration of disc space height and segmental lordosis represents a primary objective of lumbar interbody fusion (LIF), as the literature indicates that insufficient correction increases the risk of adjacent segment disease (ASD) and revision surgery [39,40].

Multiple factors affect the degree of correction achievable during LIF, including the operative approach, cage position, cage height, and lordotic angle [1,41-43]. These considerations support the development of adaptable interbody systems that accommodate patient-specific anatomy and alignment.

This study evaluated a novel modular cage system consisting of stacked internal lamellar components. The internal architecture of the construct permitted modification of its height while maintaining a consistent external footprint. Finite element modeling was performed for both compact and expanded configurations, as well as for a simplified lumbar spine-cage assembly.

Under standardized material, nominal loading, contact, and meshing conditions, the compact configuration demonstrated lower maximum von Mises stress, lower global displacement, and a higher minimum safety factor than the 18 mm configuration. The expanded construct nevertheless preserved structural continuity but developed greater equivalent and compressive principal stresses in localized load transfer regions. These findings suggest that increasing the vertical span and number of modular elements may be associated with a more mechanically demanding response under the simulated nominal pressure conditions. However, because the same pressure was applied over configuration-specific selected surfaces, the total reaction forces were not identical. The findings should therefore be interpreted as a comparison under equivalent nominal pressure rather than as an isolated evaluation of cage geometry under force-normalized loading [28,30,31,33].

When evaluated within the lumbar spine-cage assembly, the construct demonstrated a markedly different biomechanical response from that observed in the isolated cage models. Although overall displacement was greater, there was no proportional increase in peak implant stress. This finding suggests that the surrounding vertebral bodies contribute to load distribution throughout the integrated system. Therefore, it is important to evaluate an interbody device not only as an isolated structure but also in relation to the stiffness, geometry, and material characteristics of the surrounding vertebrae [34]. Isolated models are effective for illustrating configuration-dependent localized stress concentrations, whereas integrated models provide additional insight into load transfer between the implant and the surrounding bone, as well as global construct compliance.

Subsidence is one of the most significant complications of lumbar interbody fusion. Reported rates vary widely depending on the diagnostic criteria used to define subsidence, the imaging methods, the surgical technique, and the patient population [25,41-43]. Subsidence has been associated with poor bone quality, excessive restoration of disc space height, tall or narrow cages, posterior cage placement, and endplate damage [21-24,40]. Severe subsidence may lead to loss of correction, recurrence of symptoms, altered sagittal alignment, and pseudarthrosis [9,10,41,42]. Accordingly, cage design should aim to minimize localized areas of high force concentration by maximizing contact with the vertebral endplates and reducing focal loading, particularly in patients with poor bone quality.

Anatomically contoured terminal components were used to improve endplate conformity. Serrated surface features were incorporated to enhance initial mechanical engagement between the implant and bone. However, surface geometry may also contribute to localized stress concentrations. The potential benefits of enhanced resistance to migration should therefore be balanced against the risk of localized endplate loading [26].

Material selection is another important consideration in the development of future iterations of the design. PEEK offers advantages in terms of radiolucency and an elastic modulus closer to that of bone than conventional solid titanium. However, its limited osseointegration has been associated with fibrous tissue formation at the implant-bone interface, subsidence, and pseudarthrosis in several studies [44]. Biodegradable interbody cages have also been investigated as potential alternatives, although their long-term biomechanical and clinical roles remain under evaluation [45]. Experimental studies comparing interbody materials have demonstrated that porous titanium architectures may improve bone ongrowth, ingrowth, and implant-bone integration relative to conventional titanium and PEEK constructs [46-48].

The preservation of a stable footprint regardless of cage height may represent an advantageous feature of the present design. A larger cage contact area has been associated with improved load distribution and greater resistance to endplate collapse [49]. Cage geometry and peripheral endplate support may also influence construct stability during cyclic loading [50]. However, the present study demonstrates that maintaining the same footprint does not completely eliminate the additional mechanical demands associated with increased cage height. Future optimization should therefore consider not only the contact area but also the internal lamellar architecture, axial locking mechanism, and geometric transition zones.

Porous cage architectures with interconnected pore networks can promote both bone ingrowth and bone ongrowth, thereby contributing to biological fixation and mechanical stability [51]. These findings support the consideration of porous or surface-modified materials in future iterations of the present construct. Future computational comparisons of alternative materials should use identical geometries, equivalent loading conditions, comparable mesh settings, and standardized boundary conditions.

The modular architecture may also reduce endplate damage during implantation and provide greater flexibility in deformity correction, as suggested by previous biomechanical evaluations of modular cage systems [52]. Conceptually, the present design resembles expandable interbody devices in its ability to restore disc height while maintaining the geometry of the terminal components. Expandable devices may provide greater immediate restoration of disc and foraminal height; however, superior clinical outcomes have not been consistently demonstrated [53]. The present results should therefore not be interpreted as evidence of clinical superiority. Rather, they illustrate that increased reconstructive height may be achievable at the expense of greater localized mechanical demand.

Patient-specific and computer-aided cage development represents another potential direction for future optimization. Personalized implant design may improve anatomical conformity and allow cage geometry to be adapted to individual alignment and biomechanical requirements [54]. Systematic reviews have demonstrated the feasibility of both patient-specific and off-the-shelf three-dimensional-printed spinal implants [55]. Customized implants have also been used for anterior lumbar interbody fusion and complex vertebral reconstruction [56,57], while three-dimensional printing may support surgical planning and implant development in complex spinal pathologies [58]. Nevertheless, these approaches require further biomechanical and clinical validation before broader implementation.

Previous computational studies provide additional context for the present findings. Li et al. demonstrated that excessive cage height may increase endplate loading in stand-alone oblique lumbar interbody fusion constructs [59]. Wu et al. showed that cage dimensions influence endplate and instrumentation stresses in three-dimensional-printed lumbar cages [60]. El Bojairami et al. further demonstrated that cage geometry and positioning affect lordosis restoration and load transfer behavior [61]. Banerjee et al. reported that cage surface geometry may alter localized von Mises stress and spinal range of motion [62]. More broadly, integrated biomechanical models have demonstrated that load transfer depends on the interaction among the cage, vertebral endplates, and surrounding spinal structures [63]. Polikeit et al. also emphasized the influence of cancellous bone density and cage-related parameters on stress distribution following interbody cage insertion [64]. Collectively, these studies support the importance of evaluating both isolated implant mechanics and integrated vertebra-implant behavior.

Biomechanical studies of expandable devices have reported stability comparable to static constructs while also demonstrating improved restoration of disc or foraminal height [65]. However, excessive expansion or distraction may increase mechanical loading of the vertebral endplates [66]. These findings are conceptually consistent with the present results, in which the taller configuration provided greater reconstructive capacity but exhibited increased localized mechanical demand (Table 2).

**Table 2:** Selected finite element and biomechanical studies relevant to the present modular cage design

Study	Main finding	Relevance to the present study
Chayer et al. [28]	Greater cage height and suboptimal positioning increased endplate stress, whereas longer cages reduced stress and displacement.	Supports the finding that the 18 mm construct exhibited a more mechanically demanding profile than the 8 mm configuration.
Calvo-Echenique et al. [30]	Cage dimensions, endplate congruence, and positioning were major determinants of subsidence-related bone failure.	Reinforces the importance of implant geometry and implant-endplate interaction in the present modular design.
Li et al. [59]	Excessive cage height increased endplate stress, whereas moderate cage height and central positioning improved stability.	Consistent with the lower stress and displacement observed in the compact 8 mm model.
Yuan et al. [49]	A larger cage contact area increased resistance to subsidence.	Supports the potential biomechanical value of preserving a consistent footprint across different cage heights.
El Bojairami et al. [61]	Cage geometry and positioning influenced lordosis restoration, subsidence-related mechanics, and cage stress.	Highlights the importance of geometric optimization in modular cage development.
Banerjee et al. [62]	Cage surface geometry influenced load transfer, with corrugated surfaces producing greater von Mises stress than flat surfaces.	Relevant to the serrated contact surfaces of the present construct and their potential effect on local stress concentration.
Wu et al. [60]	Larger three-dimensional-printed cages reduced endplate and instrumentation stresses.	Supports the role of cage contact area and footprint in improving load distribution.
Polikeit et al. [64]	Bone density and implant-related parameters strongly influenced load transfer behavior following interbody cage insertion.	Important for interpreting the lumbar spine-cage assembly results and implant-bone interaction.
Godzik et al. [65] and Gonzalez-Blohm et al. [66]	Expandable constructs improved height restoration and provided adequate stability but could increase mechanical loading of the vertebral endplates.	Conceptually aligned with the more mechanically demanding response of the 18 mm configuration.
Wang and Wu [34]	Finite element analysis provides a useful framework for evaluating interbody cage geometry, materials, and biomechanical behavior.	Supports the methodological relevance of the finite element analyses performed in the present study.

There are several limitations inherent in the finite element analyses described above. First, the analyses were developed using simplified linear static models and therefore did not account for cyclic loading, fatigue, impact loading, creep, or other time-dependent phenomena. Consequently, the data reflect the initial structural response under controlled compressive conditions rather than long-term implant durability.

Second, idealized contact and boundary conditions were assumed. Bonded interfaces and fixed or frictionless constraints enable computationally stable simulations; however, these assumptions do not accurately reproduce physiological implant-bone micromotion or the complex constraint interactions present within an intact spinal motion segment. Furthermore, the facet joints, spinal ligaments, paraspinal muscles, posterior instrumentation, and patient-specific endplate geometry were not included in the integrated model.

Third, simplified homogeneous material properties were assigned to the vertebral components. Biological bone is heterogeneous and anisotropic and varies regionally and between individuals in terms of density and stiffness. Therefore, the current model cannot reliably estimate patient-specific endplate failure or subsidence risk.

Fourth, the isolated cage configurations were assigned identical PEEK material properties, nominal pressure magnitudes, contact definitions, and mesh-generation parameters. However, pressure was applied to configuration-specific selected surfaces, resulting in different total reaction forces. Therefore, although the influence of differing material definitions was eliminated, the observed differences cannot be attributed exclusively to implant height or geometry under identical resultant loading. Future analyses should apply an equivalent total force or otherwise normalize loading according to the effective loaded surface area.

Fifth, no formal mesh-convergence or mesh-independence analysis was performed. Consequently, the sensitivity of the reported stress and displacement values to element density remains uncertain. This limitation is particularly relevant to localized peak stresses and contact pressures near geometric transitions and component interfaces. Although identical mesh-generation parameters were used for the comparative models, this standardization does not itself establish convergence. Future analyses should incorporate progressively refined meshes and predefined convergence criteria based on global displacement and both peak and regionally averaged stress measures.

No experimental biomechanical testing, cadaveric validation, wear assessment, or in vivo evaluation was performed. Therefore, these findings illustrate mechanically plausible behavior within simplified computational models but do not establish clinical safety, fusion performance, resistance to subsidence, or superiority over existing interbody devices. Further investigations should include sensitivity analyses, physiologically representative loading conditions, improved biological material representations, experimental validation, and direct comparisons with established cage designs.

## Conclusions

The proposed modular cage-like prosthetic construct demonstrated configuration-dependent mechanical behavior under standardized material, nominal pressure, contact, and meshing conditions. The compact 8 mm configuration exhibited lower equivalent von Mises stress, lower global displacement, and a higher minimum safety factor than the expanded 18 mm configuration. The 18 mm construct maintained structural continuity but developed greater localized equivalent and compressive stresses.

These findings provide an initial computational basis for further optimization of the modular architecture, particularly the load-transfer regions of the expanded construct. However, the results remain preliminary and require confirmation using standardized material properties and loading conditions, more physiologically representative spinal models, experimental biomechanical testing, and cadaveric or in vivo validation before any conclusions regarding clinical applicability can be made.
